# Supplementary material for: Benzo[A]Pyrene Biodegradation by Multiple and Individual Mesophilic Bacteria under Axenic Conditions and in Soil Samples
Source: Int J Environ Res Public Health. 2023 Jan 19;20(3):1855. doi: 10.3390/ijerph20031855 (PMC9914810; doi:10.3390/ijerph20031855)
Supplement: Supplementary file 1 [file ijerph-20-01855-s001.zip › Figure S1_.pptx]

## Slide 1
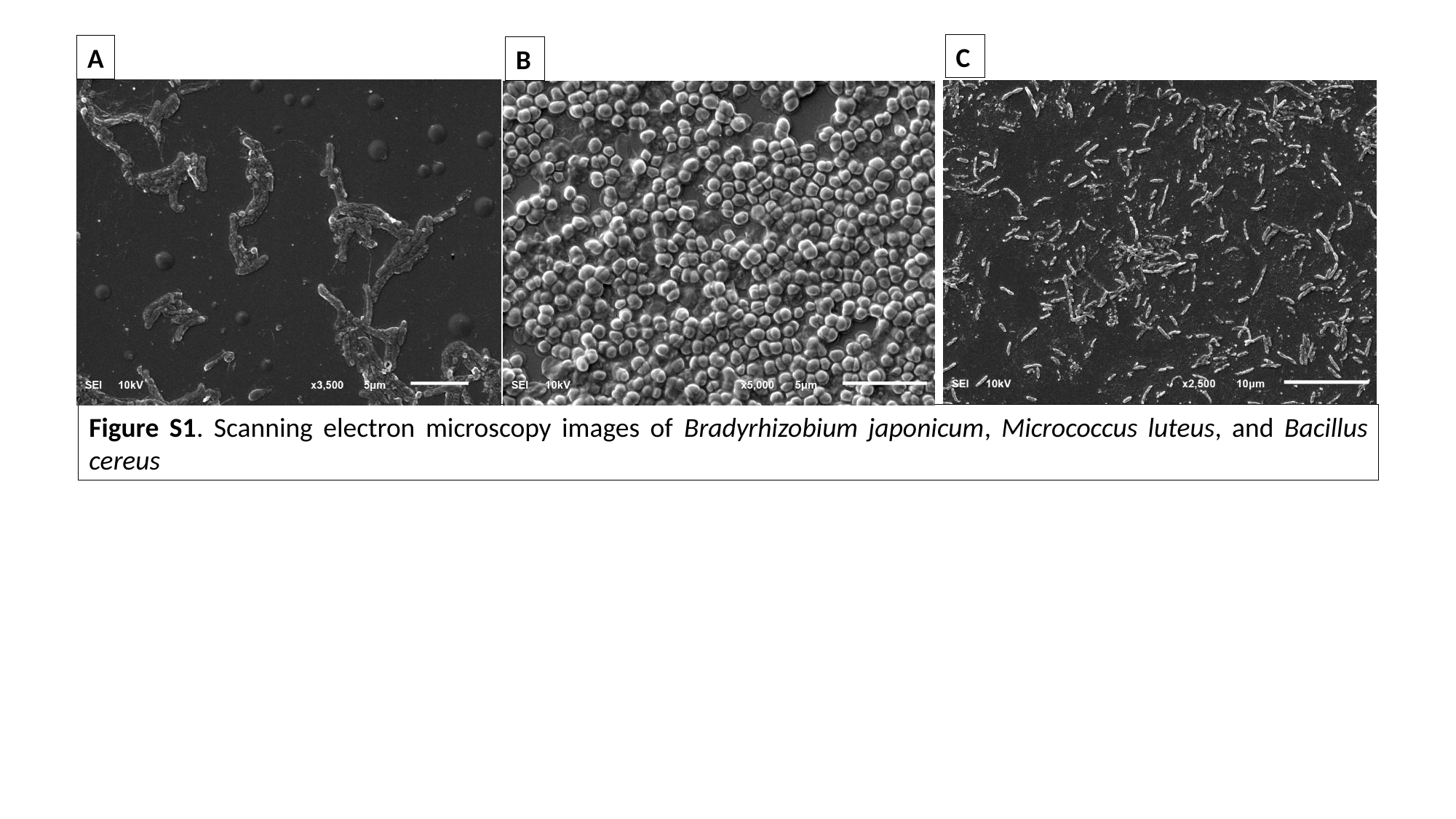

C
A
B
Figure S1. Scanning electron microscopy images of Bradyrhizobium japonicum, Micrococcus luteus, and Bacillus cereus
