# Supplementary material for: Benzo[A]Pyrene Biodegradation by Multiple and Individual Mesophilic Bacteria under Axenic Conditions and in Soil Samples
Source: Int J Environ Res Public Health. 2023 Jan 19;20(3):1855. doi: 10.3390/ijerph20031855 (PMC9914810; doi:10.3390/ijerph20031855)
Supplement: Supplementary file 1 [file ijerph-20-01855-s001.zip › Figure S2 Metabolite identification.pdf]

Figure S2: Mass spectrometry of benzo[a]pyrene and its metabolites in three individual bacterial strains and their consortium

JBZ5E:

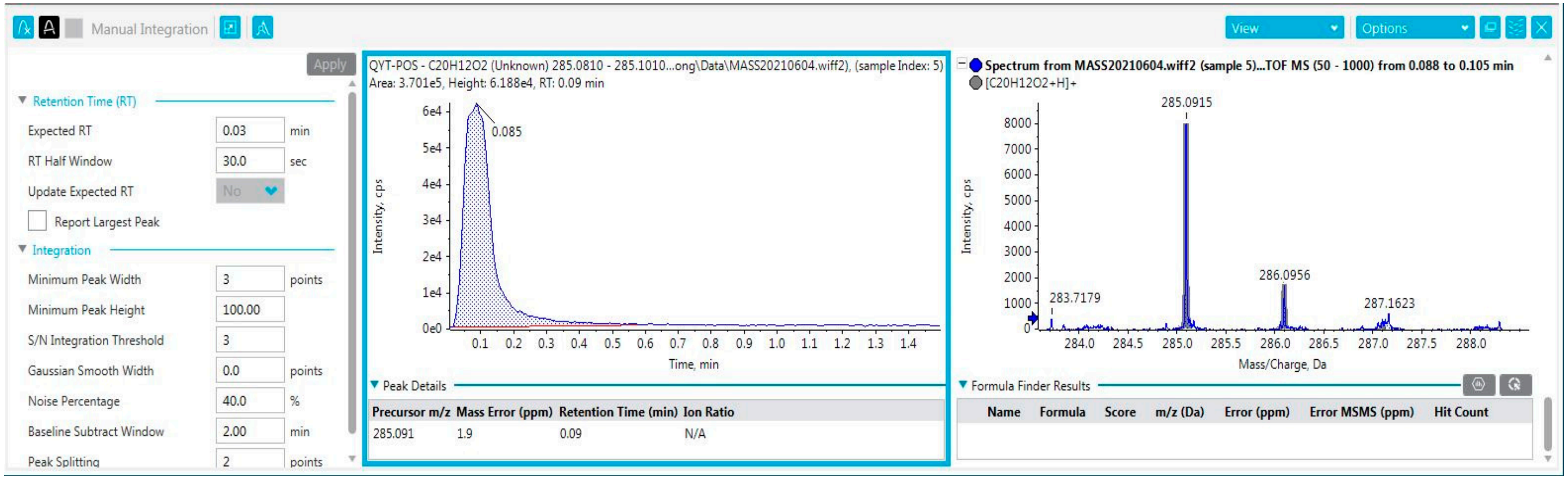

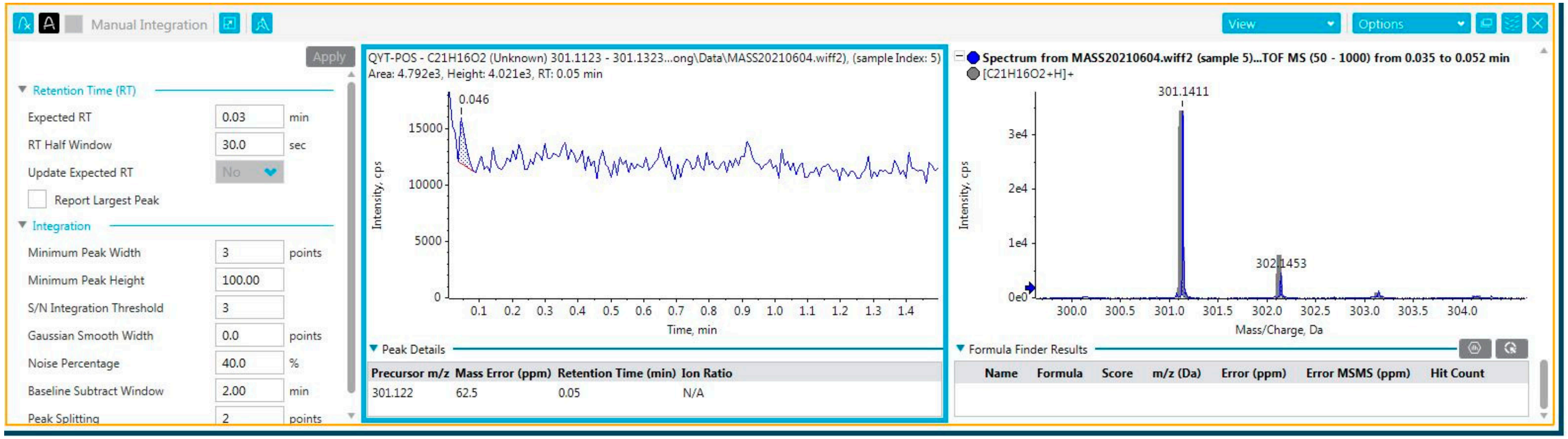

JBZ2B:

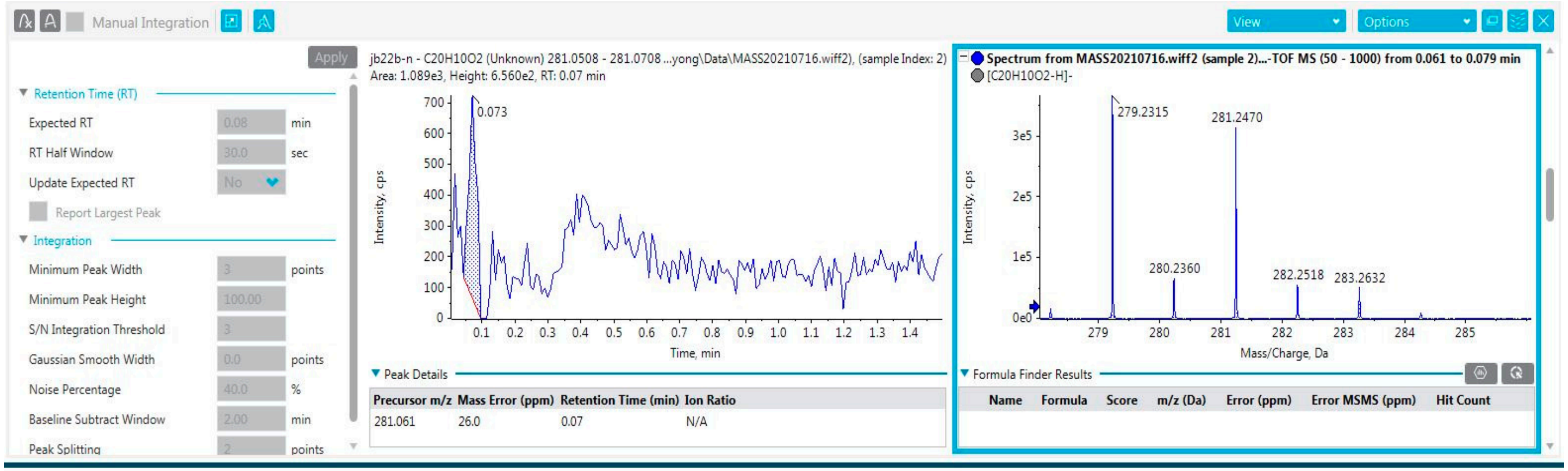

Manual Integration
Apply
jb22b-n - C20H10O2 (Unknown) 281.0508 - 281.0708 ...yong\Data\MASS20210716.wiff2), (sample Index: 2)
Area: 1.089e3, Height: 6.560e2, RT: 0.07 min
0.073
Spectrum from MASS20210716.wiff2 (sample 2)...-TOF MS (50 - 1000) from 0.061 to 0.079 min
[C20H10O2-H]-
279.2315
281.2470
280.2360
282.2518
283.2632
Precursor m/z Mass Error (ppm) Retention Time (min) Ion Ratio
281.061 26.0 0.07 N/A

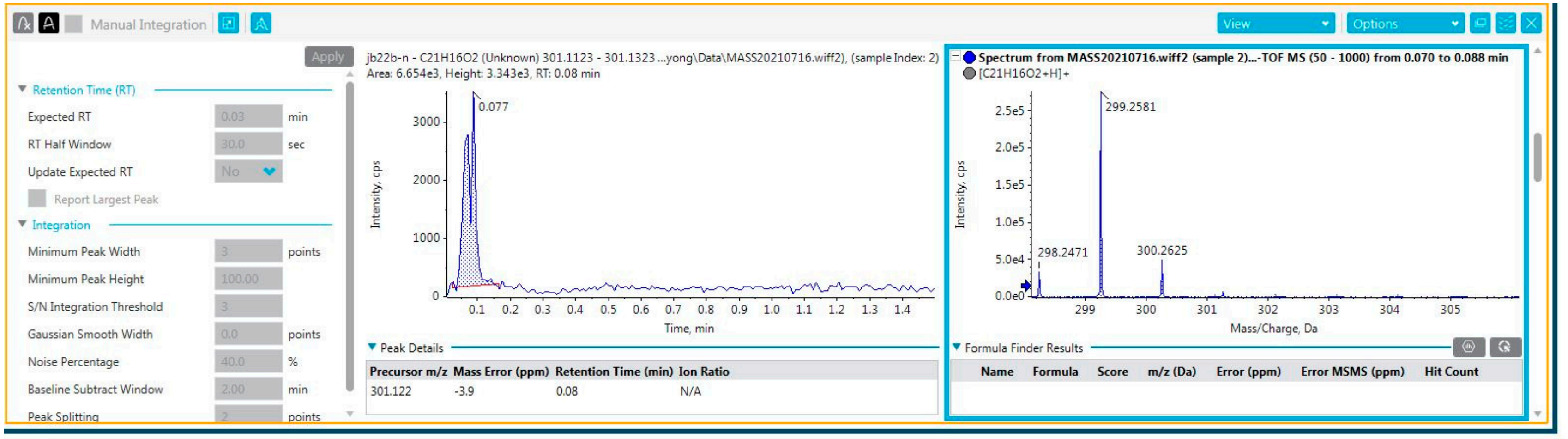

Manual Integration
Apply
jb22b-n - C21H16O2 (Unknown) 301.1123 - 301.1323 ...yong\Data\MASS20210716.wiff2), (sample Index: 2)
Area: 6.654e3, Height: 3.343e3, RT: 0.08 min
0.077
Spectrum from MASS20210716.wiff2 (sample 2)...-TOF MS (50 - 1000) from 0.070 to 0.088 min
[C21H16O2+H]+
299.2581
298.2471
300.2625
Precursor m/z Mass Error (ppm) Retention Time (min) Ion Ratio
301.122 -3.9 0.08 N/A

Con：

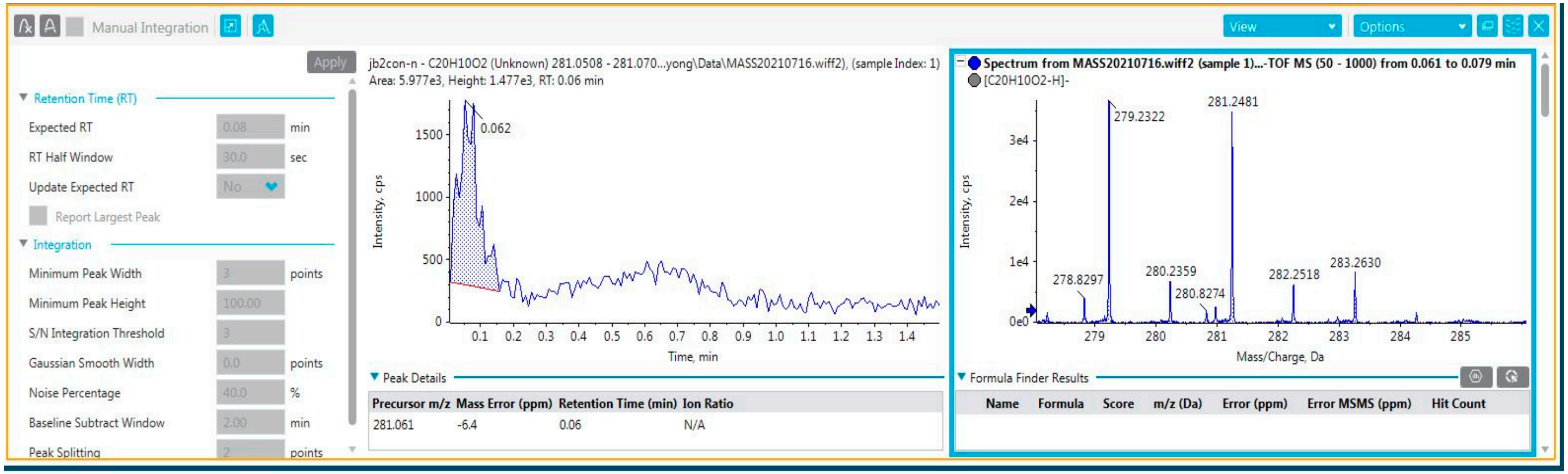

Manual Integration
View
Options
Apply
Retention Time (RT)
Expected RT 0.08 min
RT Half Window 30.0 sec
Update Expected RT No
Report Largest Peak
Integration
Minimum Peak Width 3 points
Minimum Peak Height 100.00
S/N Integration Threshold 3
Gaussian Smooth Width 0.0 points
Noise Percentage 40.0 %
Baseline Subtract Window 2.00 min
Peak Splitting 2 points
jb2con-n - C20H10O2 (Unknown) 281.0508 - 281.070...yong\Data\MASS20210716.wiff2), (sample Index: 1)
Area: 5.977e3, Height: 1.477e3, RT: 0.06 min
0.062
Intensity, cps
Time, min
Peak Details
Precursor m/z Mass Error (ppm) Retention Time (min) Ion Ratio
281.061 -6.4 0.06 N/A
Spectrum from MASS20210716.wiff2 (sample 1)...-TOF MS (50 - 1000) from 0.061 to 0.079 min
[C20H10O2-H]-
279.2322
281.2481
278.8297
280.2359
280.8274
282.2518
283.2630
Intensity, cps
Mass/Charge, Da
Formula Finder Results
Name Formula Score m/z (Da) Error (ppm) Error MSMS (ppm) Hit Count

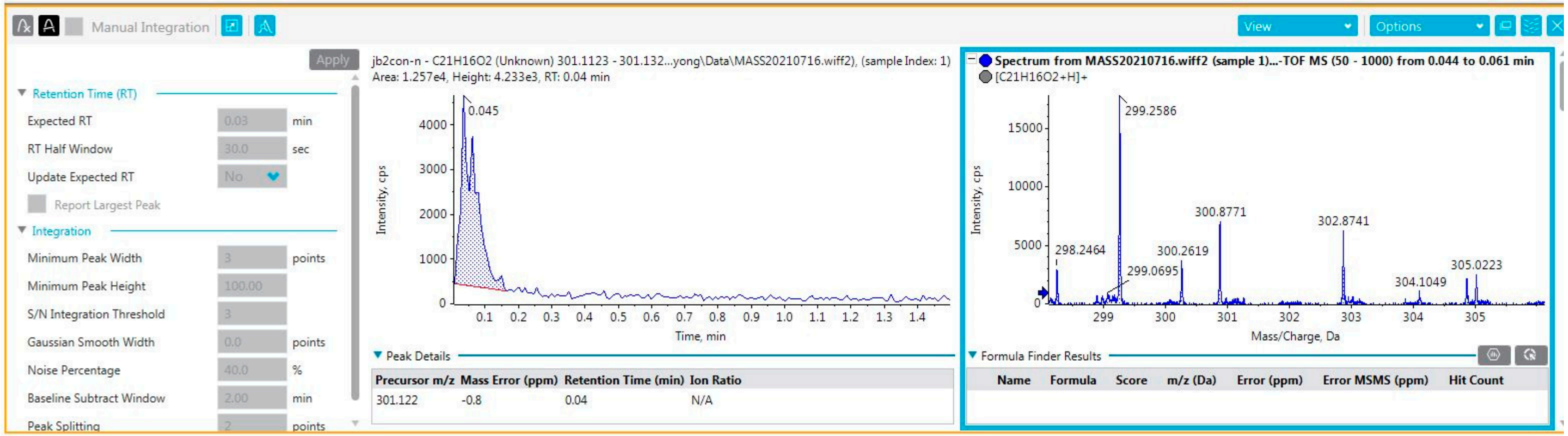

Manual Integration
Apply
Retention Time (RT)
Expected RT
RT Half Window
Update Expected RT
Report Largest Peak
Integration
Minimum Peak Width
Minimum Peak Height
S/N Integration Threshold
Gaussian Smooth Width
Noise Percentage
Baseline Subtract Window
Peak Splitting
jb2con-n - C21H16O2 (Unknown) 301.1123 - 301.132...yong\Data\MASS20210716.wiff2), (sample Index: 1)
Area: 1.257e4, Height: 4.233e3, RT: 0.04 min
Intensity, cps
Time, min
Peak Details
Precursor m/z Mass Error (ppm) Retention Time (min) Ion Ratio
301.122 -0.8 0.04 N/A
Spectrum from MASS20210716.wiff2 (sample 1)...-TOF MS (50 - 1000) from 0.044 to 0.061 min
[C21H16O2+H]+
Mass/Charge, Da
Formula Finder Results
Name Formula Score m/z (Da) Error (ppm) Error MSMS (ppm) Hit Count

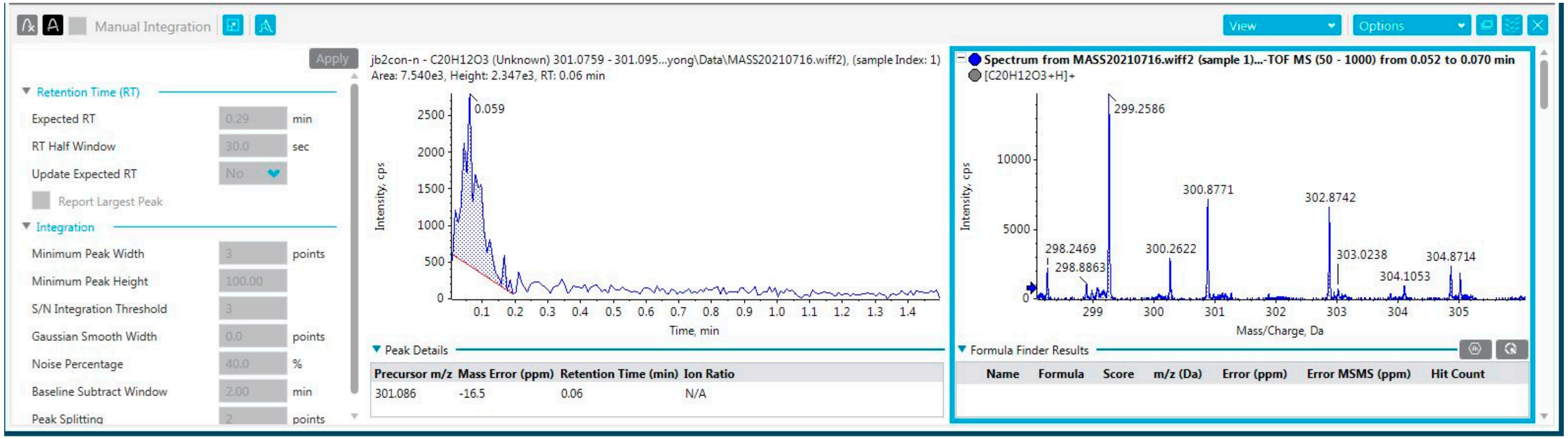

Manual Integration
Apply
jb2con-n - C20H12O3 (Unknown) 301.0759 - 301.095...yong\Data\MASS20210716.wiff2), (sample Index: 1)
Area: 7.540e3, Height: 2.347e3, RT: 0.06 min
Peak Details
Precursor m/z Mass Error (ppm) Retention Time (min) Ion Ratio
301.086 -16.5 0.06 N/A
Spectrum from MASS20210716.wiff2 (sample 1)...-TOF MS (50 - 1000) from 0.052 to 0.070 min
[C20H12O3+H]+
Formula Finder Results

JBZ1A:

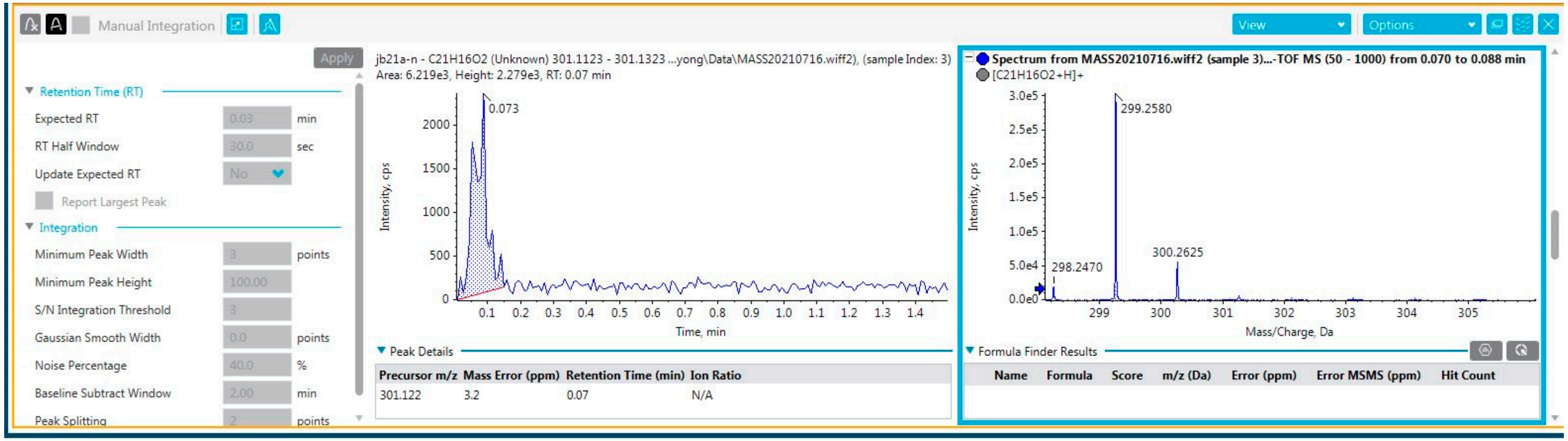

Manual Integration
View
Options
Apply
Retention Time (RT)
Expected RT
0.03
min
RT Half Window
30.0
sec
Update Expected RT
No
Report Largest Peak
Integration
Minimum Peak Width
3
points
Minimum Peak Height
100.00
S/N Integration Threshold
3
Gaussian Smooth Width
0.0
points
Noise Percentage
40.0
%
Baseline Subtract Window
2.00
min
Peak Splitting
2
points
jb21a-n - C21H16O2 (Unknown) 301.1123 - 301.1323 ...yong\Data\MASS20210716.wiff2), (sample Index: 3)
Area: 6.219e3, Height: 2.279e3, RT: 0.07 min
0.073
Intensity, cps
Time, min
Peak Details
Precursor m/z
Mass Error (ppm)
Retention Time (min)
Ion Ratio
301.122
3.2
0.07
N/A
Spectrum from MASS20210716.wiff2 (sample 3)...-TOF MS (50 - 1000) from 0.070 to 0.088 min
[C21H16O2+H]+
299.2580
300.2625
298.2470
Intensity, cps
Mass/Charge, Da
Formula Finder Results
Name
Formula
Score
m/z (Da)
Error (ppm)
Error MSMS (ppm)
Hit Count
